# Transient hyperlactatemia during intravenous administration of glycerol: a prospective observational study

**DOI:** 10.1186/s40560-018-0323-7

**Published:** 2018-08-28

**Authors:** Shinshu Katayama, Ken Tonai, Yuya Goto, Kansuke Koyama, Toshitaka Koinuma, Jun Shima, Masahiko Wada, Shin Nunomiya

**Affiliations:** Division of Intensive Care, Department of Anesthesiology and Intensive Care Medicine, Jichi Medical University School of Medicine, 3311-1, Yakushiji, Shimotsuke, Tochigi 329-0498 Japan

**Keywords:** Glycerol, Hyperlactatemia, Intensive care unit, Mannitol

## Abstract

**Background:**

Intravenous glycerol treatment, usually administered in the form of a 5% fructose solution, can be used to reduce intracranial pressure. The administered fructose theoretically influences blood lactate levels, although little is known regarding whether intravenous glycerol treatment causes transient hyperlactatemia. This study aimed to evaluate blood lactate levels in patients who received intravenous glycerol or mannitol.

**Methods:**

This single-center prospective observational study was performed at a 14-bed general intensive care unit between August 2016 and January 2018. Patients were excluded if they were < 20 years old or had pre-existing hyperlactatemia (blood lactate > 2.0 mmol/L). The included patients received intravenous glycerol or mannitol to reduce intracranial pressure and provided blood samples for lactate testing before and after the drug infusion (before the infusion and after 15 min, 30 min, 45 min, 60 min, 90 min, 120 min, and 150 min).

**Results:**

Among the 33 included patients, 13 patients received 200 mL of glycerol over 30 min, 13 patients received 200 mL of glycerol over 60 min, and 7 patients received 300 mL of mannitol over 60 min. Both groups of patients who received glycerol had significantly higher lactate levels than the mannitol group (2.8 mmol/L vs. 2.2 mmol/L vs. 1.6 mmol/L, *P* < 0.0001), with the magnitude of the increase in lactate levels corresponding to the glycerol infusion time. There were no significant inter-group differences in cardiac index, stroke volume, or stroke volume variation. In the group that received the 30-min glycerol infusion, blood lactate levels did not return to the normal range until after 120 min.

**Conclusions:**

Intravenous administration of glycerol leads to higher blood lactate levels that persist for up to 120 min. Although hyperlactatemia is an essential indicator of sepsis and/or impaired tissue perfusion, physicians should be aware of this phenomenon when assessing the blood lactate levels.

## Background

Lactate is a sensitive indicator of tissue perfusion and metabolism that helps quantify the balance between aerobic and anaerobic metabolism [1–3]. In addition, lactate is correlated with intravascular volume and sepsis status and is associated with circulatory shock severity [4–6]. In this context, lactate levels of > 2.0 mmol/L are included in the Sepsis-3 criteria for septic shock [7], and lactate level measurements are widely recognized as important in the critical care setting. In clinical practice, it is recommended that attempts be made to increase cardiac output using fluid resuscitation or inotropes, which is called lactate-guided resuscitation [8–11].

Intravenous glycerol treatment can be used to reduce intracranial pressure and is usually administered in the form of a 5% fructose solution with concentrated glycerin. Despite low-quality evidence of the efficacy [12, 13], glycerol is widely used in Japan for patients with acute stroke or brain trauma, without significant side effects [14]. Although children with some congenital metabolic diseases experience hyperlactatemia (i.e., congenital glycerin metabolic disorders), little is known about intravenous glycerol-induced hyperlactatemia [15]. However, fructose can theoretically influence lactate metabolism [16, 17], and lactate levels might change during the administration of glycerol, which could lead to a misdiagnosis or overestimation of disease severity. Therefore, the present study evaluated the blood lactate levels in patients who received intravenous glycerol or mannitol and whether the rate of the intravenous glycerol infusion influenced blood lactate concentrations.

## Methods

This single-center, prospective, observational study was performed at a 14-bed general intensive care unit in a university hospital (Tochigi, Japan). The study protocol was approved by the institutional ethics committee of Jichi Medical University Hospital (16-120). Written informed consent was obtained from the participants or their nearest relatives.

### Participants

This study included patients who were admitted to the intensive care unit and received intracranial pressure-reducing therapy using intravenous glycerol or mannitol between August 2016 and January 2018. Patients were excluded if they were < 20 years old, had an end-stage renal failure and were receiving chronic dialysis, had hepatic dysfunction, or had abnormal lactate levels (> 2.0 mmol/L) before starting the intracranial pressure-reducing therapy.

### Administration of glycerol or mannitol

The intravenous glycerol (GLYCEREB®; Terumo, Japan) was administered at a volume of 200 mL over 30 min or 60 min. The intravenous mannitol (20% MANNITOL INJECTION®; Yoshindo, Japan) was administered at a volume of 300 mL over 60 min. The selection of the drug and infusion rate was made by the attending physician.

### Lactate measurement

Blood samples were obtained for lactate measurements at eight time points (before the infusion and after 15 min, 30 min, 45 min, 60 min, 90 min, 120 min, and 150 min) and were immediately transferred to the laboratory for testing. The blood gas analyses, which included lactate measurements, were performed using an ABL800 FLEX device (Radiometer Medical ApS, Denmark) or a RAPIDLAB1265 device (Siemens Healthcare Diagnostics Inc., Tarrytown, NY, USA).

### Data collection

The following information was collected for all patients: age, sex, body weight, body height, present disease, Acute Physiology and Chronic Health Evaluation II (APACHE II) score [18], and Sequential Organ Failure Assessment (SOFA) score [19]. We also recorded data regarding the requirement for mechanical ventilation and the 28-day survival status. A FloTrac device (version 1.5, Edwards LifeSciences, Irvine, USA) was used to measure cardiac index (CI), stroke volume index (SVI), and stroke volume variation (SVV).

### Statistical analysis

Variables were compared among these groups using Pearson’s chi-square test, the Kruskal-Wallis test, or the Steel-Dwass test as appropriate. All analyses were performed using JMP software (version 13; SAS Institute Inc., Cary, NC, USA). Data were presented as median and interquartile range or as number and percentage. Differences were considered statistically significant at *P* values of < 0.05.

## Results

### Enrollment and baseline characteristics

During the study period, 37 patients were enrolled and 4 patients were excluded based on the pre-existing high lactate levels (> 2.0 mmol/L). The characteristics of the 33 included patients are summarized in Table 1. Thirteen patients received 200 mL of glycerol over 30 min, 13 patients received 200 mL of glycerol over 60 min, and 7 patients received 300 mL of mannitol over 60 min. Although the groups’ APACHE II and SOFA scores were not significantly different, the group that received a 30-min glycerol infusion had a significantly lower 28-day survival rate compared to the other two groups (69.2% vs. 100% vs. 100%; *P* = 0.03). Table 2 shows the laboratory findings from before the start of the infusions, with no evidence of significant inter-group differences. There were no significant differences in parameters of hepatic function between the groups.

**Table 1:** Patient characteristics

	Glycerol 200 mL/30 min	Glycerol 200 mL/60 min	Mannitol 300 mL/60 min	*P* value
	*n* = 13	*n* = 13	*n* = 7	
Age, years	57 (47–66)	46 (38–69)	58 (45–62)	0.592
Male, sex	6 (46.1%)	5 (38.5%)	4 (57.2%)	0.724
Height, cm	161 (154–166)	155 (154–164)	170 (155–170)	0.229
Body weight, kg	60 (51–65)	60 (55–69)	62 (54–67)	0.731
Body mass index, kg/m^2^	23.9 (19.4–25.7)	25.0 (21.2–26.9)	21.7 (21.1–27.9)	0.573
Diseases, %				0.426
Brain tumor	7.7%	23.1%	0.0%	
Intracranial hemorrhage	15.4%	23.1%	14.3%	
Sub-arachnoid hemorrhage	23.1%	7.7%	42.9%	
Cerebral infarction	38.5%	38.5%	28.6%	
Traumatic brain injury	0.0%	7.7%	14.3%	
Other	15.4%	0.0%	0.0%	
IHD	7.7%	0.0%	0.0%	0.452
CHF	0.0%	15.4%	0.0%	0.194
Atrial fibrillation	15.4%	15.4%	0.0%	0.542
Diabetes mellitus	15.4%	23.1%	14.3%	0.840
Hypertension	30.8%	23.1%	42.9%	0.655
APACHE II	19 (13–22)	16 (13–28)	18 (11–22)	0.858
28-day survival, %	69.2%	100.0%	100.0%	0.030

*APACHE II* Acute Physiology and Chronic Health Evaluation II score, *CHF* chronic heart failure, *IHD* ischemic heart disease

**Table 2:** Laboratory findings immediately before the administration of intracranial pressure-reducing agents

	Glycerol 200 mL/30 min	Glycerol 200 mL/60 min	Mannitol 300 mL/60 min	*P* value
WBC, 10^9^/L	12.5 (10.2–14.7)	10.1 (9.1–12.2)	1.4 (8.1–13.4)	0.228
Hb, g/dL	9.5 (8.6–11.3)	8.2 (7.8–10.4)	9.4 (8.9–10.0)	0.256
CRP, mg/dL	8.1 (3.8–14.5)	5.7 (0.7–9.5)	5.1 (2.7–9.5)	0.271
Alb, g/dL	2.3 (2.1–2.7)	2.6 (2.3–3.2)	3.0 (2.5–3.4)	0.143
BUN, mg/dL	10 (9–13)	12 (8–19)	14 (10–16)	0.502
Creatinine, mg/dL	0.67 (0.49–0.87)	0.51 (0.43–1.11)	0.61 (0.50–0.97)	0.790
T-Bil, mg/dL	0.75 (0.47–1.08)	0.95 (0.56–1.68)	1.01 (0.67–1.24)	0.420
AST, U/L	28 (18–48)	26 (21–37)	29 (23–83)	0.634
ALT, U/L	18 (13–37)	19 (14–43)	20 (15–57)	0.699
LDH, U/L	249 (182–328)	231 (166–269)	216 (182–246)	0.530
P, mg/dL	2.6 (2.0–2.8)	2.8 (1.9–3.3)	2.7 (2.2–3.1)	0.828
ChE, U/L	205 (155–234)	139 (115–185)	195 (79–232)	0.211
SOFA	3 (2–5)	6 (3–7)	3 (3–4)	0.182
Mechanical ventilation	46.1%	76.9%	71.4%	0.235

*Alb* albumin, *ALT* alanine aminotransaminase, *AST* aspartate transaminase, *BUN* blood urea nitrogen, *ChE* choline esterase, *CRP* C-reactive protein, *Hb* hemoglobin, *LDH* lactate dehydrogenase, *P* phosphate, *SOFA* Sequential Organ Failure Assessment, *T-bil* total bilirubin, *WBC* white blood cells

### Comparing the groups that received glycerol or mannitol

**Fig. 1:**
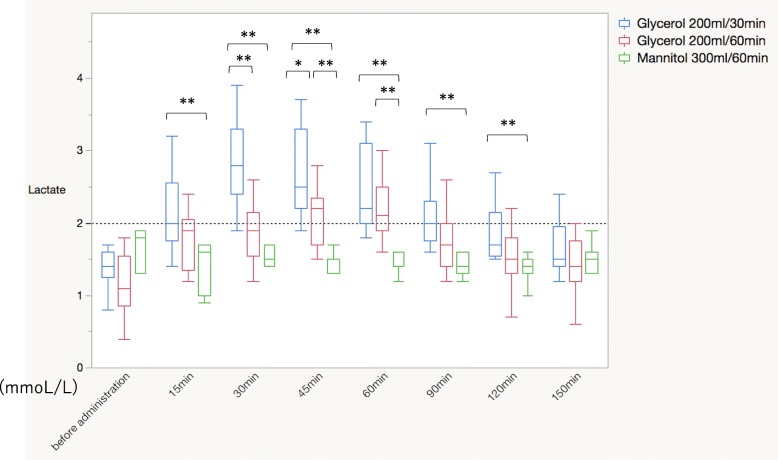
Blood lactate dynamics during the administration of glycerol or mannitol. Horizontal dot line means the upper normal limit of blood lactate level. *P < 0.05, **P < 0.01

Both groups of patients who received glycerol had significantly higher lactate levels than the mannitol group (2.8 mmol/L vs. 2.2 mmol/L vs. 1.6 mmol/L, respectively; *P* < 0.0001), with the magnitude of the increase in lactate levels corresponding to the glycerol infusion time. Compared to the mannitol group, the 30-min glycerol group had significantly higher lactate levels at all measured time points between 15 and 120 min, and all of these patients had high lactate levels (> 2.0 mmol/L) during the infusions. The 60-min glycerol group had significantly higher lactate levels at the 45-min and 60-min measurements, relative to the mannitol group, and 9 patients (69.2%) in the 60-min glycerol group had high lactate levels during the infusions. None of the patients in the mannitol group had high lactate levels during the infusions (100% vs. 69.2% vs. 0.0%, respectively; *P* < 0.0001) (Fig. 1). None of the patients exhibited signs of intravenous hemolysis.

### Hemodynamics and blood gas analyses

**Fig. 2:**
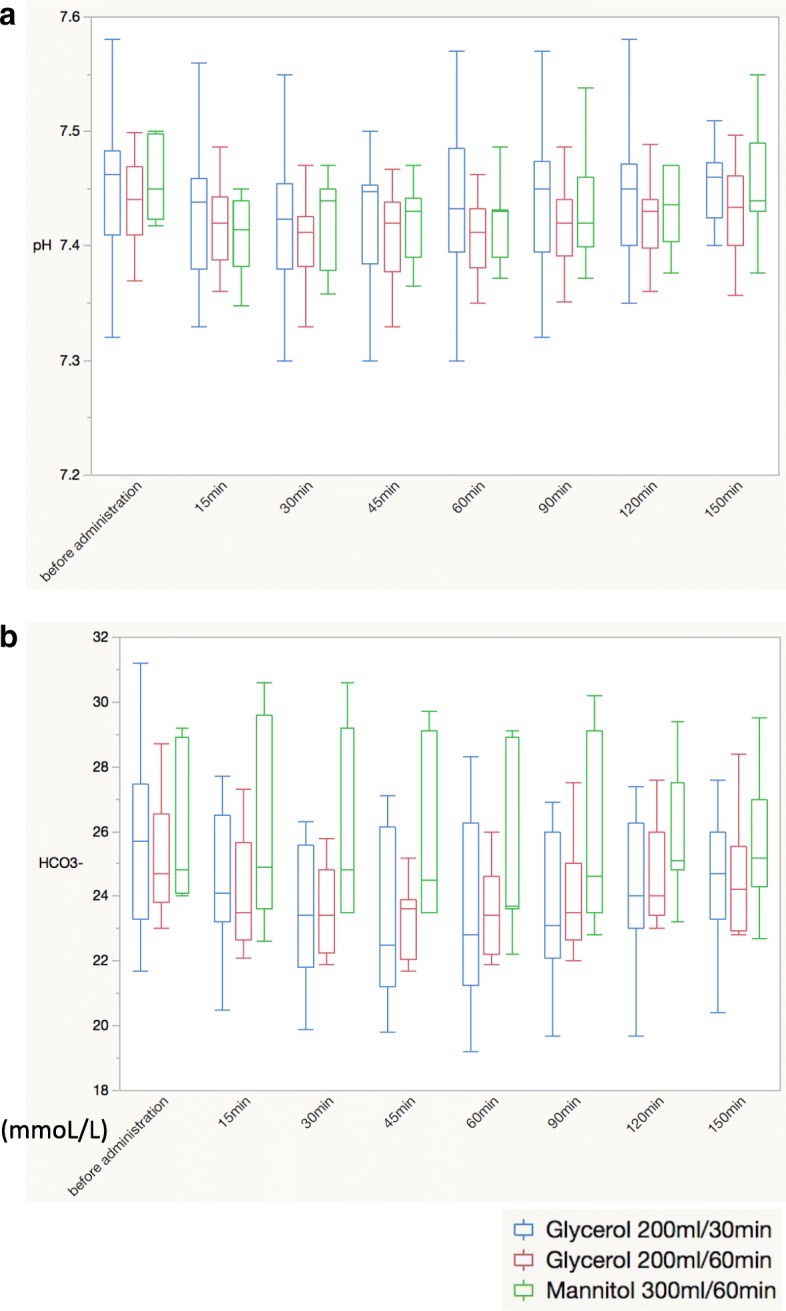
Acid-base status during the administration of glycerol or mannitol. **a** pH. **b** HCO_3_^−^

**Fig. 3:**
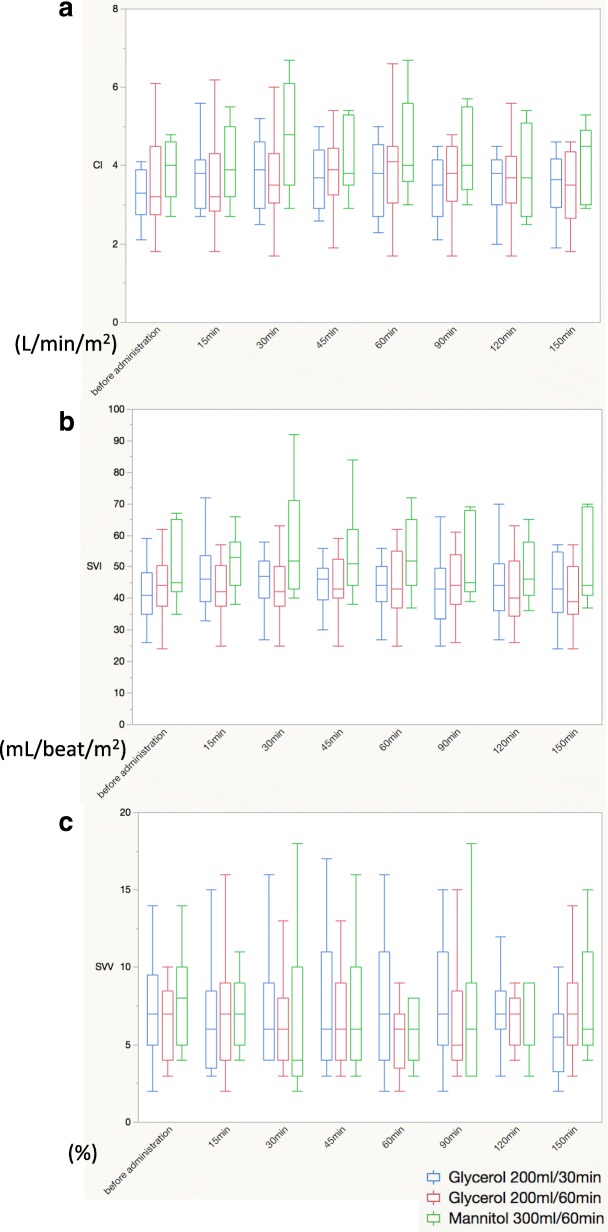
Hemodynamics during the administration of glycerol or mannitol. **a** Cardiac index. **b** Stroke volume index. **c** Stroke volume variation

During the glycerol or mannitol treatment, no significant inter-group differences were observed in terms of CI, SVI, SVV, pH, or HCO_3_^−^ levels (Figs. 2 and 3).

## Discussion

The present study revealed that intravenous glycerol treatment increased the lactate levels in all patients, and 84.6% of these patients developed lactate levels of > 2.0 mmol/L. This study also revealed that lactate levels differed significantly according to the duration of glycerol infusion. However, intravenous mannitol treatment did not influence the lactate levels, and there were no inter-group differences in cardiac output and stroke volume parameters. Therefore, the effects of osmotic diuresis seem unrelated to the increased lactate levels.

Hyperlactatemia during glycerol administration is caused by the fructose that is added to the glycerol solution. Intravenously administered fructose is metabolized by the liver and converted by fructokinase into fructose-1-phosphate, which then enters the glycolytic or gluconeogenesis cycle before being metabolized into lactate, glucose, or glycogen. Fructokinase activity remains unchanged in a state of insulin deficiency, and fructose is quickly metabolized, which causes pyruvate accumulation. This occurs even in the presence of reduced glucose tolerance because fructose metabolism does not involve glucokinase or phosphofructokinase, which has decreased the activities in an insulin-deficient state. Furthermore, metabolism of fructose into pyruvate in the liver is rapid enough to cause significant accumulation of un-metabolized pyruvate by the tricarboxylic acid cycle, which causes hyperlactatemia. Approximately 30% of the administered fructose is converted into glucose [17], with the intravenous fructose stimulating splanchnic lactate release both at rest [16] and during exercise [20], as well as glucose-stimulated extrasplanchnic lactate production [21]. Moreover, the lactate concentration increases that are induced by fructose are positively correlated with muscle glycogen resynthesis [22], which accelerates nucleic acid turnover and uric acid production, and then causes hyperlactatemia [23].

The fructose infusion rate should not exceed 0.3 g/kg/h [24], and the recommended glycerol infusion rate should not exceed 300 mL over 30 min for a 60-kg patient, as rapid glycerol administration increases the risk of intravascular hemolysis [25]. It has been shown that administering 0.5 g/kg glycerol for 90 min is superior to 30 min for reducing intracranial pressure [26]. Although the present study did not measure haptoglobin to quantify hemolysis, we did not detect any apparent signs of intravascular hemolysis among the included patients. However, we found that the increase in lactate levels was larger at higher infusion rates. Thus, physicians should be aware of this phenomenon and ideally wait for at least 120 min when assessing the illness severity in patients who received glycerol. Conversely, the glycerol infusion rate should be moderated and lactate levels tested frequently in patients with pre-existing hyperlactatemia.

Hyperlactatemia can occur under several physiological conditions, although it is often correlated with oxygen delivery, cardiac function, intravascular volume, and hemoglobin levels. For example, hemodynamically unstable patients are likely to present with increased lactate levels. In this context, agents that reduce intracranial pressure (e.g., glycerol or mannitol) induce strong osmotic diuresis that can cause reduced stroke volume and intravascular volume that lead to elevated lactate levels. However, the present study did not detect significant relationships between the various hemodynamic parameters and hyperlactatemia, and none of the patients developed active bleeding or desaturation during the administration of glycerol or mannitol. Therefore, it appears unlikely that hyperlactatemia caused by intravenous glycerol administration is related to impaired hemodynamics or oxygen delivery.

The present study has several limitations. First, this was a small single-center observational study; nevertheless, we were still able to detect a significant relationship between intravenous glycerol treatment and elevated blood lactate levels. Second, we did not evaluate whether the patients had congenital metabolic disorders, although these conditions are very rare. Finally, we did not evaluate any increases in lactate levels among patients with pre-existing hyperlactatemia (who were excluded from the study). Although the effects of glycerol on lactate levels disappeared after at least 120 min had passed in patients with normal baseline lactate levels, it is plausible that in patients with hyperlactatemia, the effects of glycerol may be prolonged past 120 min due to the impaired lactate metabolism, thus warranting a longer observation period than used in this study. Therefore, further studies are needed to evaluate blood lactate levels during glycerol administration in patients with pre-existing hyperlactatemia.

## Conclusions

Intravenous administration of glycerol induced an increase in blood lactate levels that persisted for up to 120 min. Although hyperlactatemia is an essential indicator of sepsis and/or impaired tissue perfusion, this phenomenon should be considered when assessing the blood lactate levels.

## Abbreviations

APACHE II: Acute Physiology and Chronic Health Evaluation II
CI: Cardiac index
SOFA: Sequential Organ Failure Assessment
SVI: Stroke volume index
SVV: Stroke volume variation
